# Sustainable Downscaled Catalytic Colorimetric Determination of Manganese in Freshwater Using Smartphone-Based Monitoring Oxidation of 3,3′,5,5′-Tetramethylbenzidine by Periodate

**DOI:** 10.3390/molecules27154841

**Published:** 2022-07-28

**Authors:** Sutasinee Apichai, Parichart Kummuntakoon, Thanawat Pattananandecha, Jakaphun Julsrigival, Kasirawat Sawangrat, Fumihiko Ogata, Naohito Kawasaki, Kate Grudpan, Chalermpong Saenjum

**Affiliations:** 1 Department of Pharmaceutical Sciences, Faculty of Pharmacy, Chiang Mai University, Chiang Mai 50200, Thailand; sutasinee.apichai@gmail.com (S.A.); thanawat.pdecha@gmail.com (T.P.); jakkaphun@gmail.com (J.J.); kasirawat.s@cmu.ac.th (K.S.); 2 Center of Excellence for Innovation in Analytical Science and Technology for Biodiversity-Based Economic and Society (I-ANALY-S-T_B.BES-CMU), Chiang Mai University, Chiang Mai 50200, Thailand; kgrudpan@gmail.com; 3 Department of Chemistry, Faculty of Science and Technology, Chiang Mai Rajabhat University, Chiang Mai 50300, Thailand; parichart10983@gmail.com; 4 Faculty of Pharmacy, Kindai University, 3-4-1 Kowakae, Higashi-Osaka 577-8502, Japan; ogata@phar.kindai.ac.jp (F.O.); kawasaki@phar.kindai.ac.jp (N.K.); 5 Antiaging Center, Kindai University, 3-4-1 Kowakae, Higashi-Osaka 577-8502, Japan; 6 Department of Chemistry, Faculty of Science, Chiang Mai University, Chiang Mai 50200, Thailand

**Keywords:** manganese, water monitoring, colorimetry, digital image based-procedure, smartphone, on-site analysis, United Nations Sustainable Development Goals (UN-SDGs)

## Abstract

A sustainable downscaled procedure using smartphone-based colorimetric determination of manganese (Mn(II)) was developed. This novel Mn(II) determination procedure is proposed using a simple, available microwell-plate platform and a smartphone as a detector. This approach is based on the oxidation of 3,3′,5,5′-tetramethylbenzidine (TMB) by periodate using Mn(II) as a catalyst. The catalytic kinetics of Mn(II) under different conditions was investigated to determine the optimum condition where the different catalytic activities of various concentrations of Mn(II) evince. Under the optimum condition, the bluish-green product of oxidized TMB, proportioned to the concentration of Mn(II), was monitored using a smartphone camera, and the color signals were processed using ImageJ Software. The developed procedure showed great selectivity and sensitivity as linearity ranged from 1.8 × 10^−6^ to 4.6 × 10^−5^ M (0.1 to 2.5 μg/mL). The limits of detection and quantitation were 3.6 × 10^−6^ and 1.1 × 10^−5^ M (0.2 and 0.6 μg/mL), respectively. The determination of Mn(II) in freshwater samples was demonstrated to assess environmental water quality as an initial model to more easily promote water management according to the United Nations Sustainable Development Goals (UN-SDGs). The intensity of the red could be successfully applied to evaluate Mn(II) in canals and river water with no significant differences compared with the reference method of Inductively Coupled Plasma Optical Emission Spectrometry at a confidence level of 95%.

## 1. Introduction

Rapid urbanization and human activities, such as electricity generation, transportation, fossil fuel combustion, use of various chemicals and other related activities have resulted in heavy metal pollution. Heavy metal contaminants are regarded as one of the most serious dangers to natural ecosystems because of their inability to biodegrade, instability and toxicity regarding several aquatic organisms [1]. Therefore, heavy metal analysis and treatment are necessary, and related cost-effective technology has continuously developed [2]. Manganese is a heavy metal causing acute and chronic toxicity in many aquatic species, as evidenced in the literature [3]. It has been widely used in several fields, such as applying manganese dioxide to produce dry-cell batteries, matches, fireworks, porcelain and glass-bonding materials, amethyst glass and as a precursor for other manganese compounds. In the agricultural field, manganese sulfate is used as a fertilizer in manganese-deficient soils, pesticides, fungicides and as livestock supplement. In general use and daily life, potassium permanganate is used as a disinfectant, an antialgal agent, a metal cleaner, a tanning and bleaching agent and a preservative for fresh flowers and fruits. For this reason, manganese is ubiquitous in the environment. Manganese contamination of water results from it being released or discharged from industrial facilities or as leachate from landfills and soil [4]. Because several manganese compounds are readily soluble in water, they constitute a high risk to aquatic animals through considerable exposure and their inability to survive in contaminated water.

Many of the requirements and policies regarding water management were issued to guard the environment and protect life. Goal 6 of the United Nations Sustainable Development Goals (UN-SDGs) aims at protecting and restoring water-related ecosystems by providing developing countries with capacity-building assistance in water and sanitation-related activities and initiatives such as water harvesting, desalination, water efficiency, wastewater treatment, recycling and recycling reuse technologies [5]. These include local community involvement in improving water and sanitation management. Australian and New Zealand guidelines for fresh and marine water quality defines toxicant trigger values for aquatic species; the trigger value of a manganese concentration of 1.7 mg/L has been calculated so that six tropical freshwater species in the environment could still survive at an estimated 95% [6]. The Environment Agency (UK) evaluated manganese toxicity in freshwaters based on a Species Sensitivity Distribution (SSD) of 12 toxicity estimates and advised a Predicted No Effect Concentration (PNEC) of 62 to 123 μg/L for aquatic ecosystems [7]. The Water Environment Partnership in Asia (WEPA) defines ambient standards for manganese in ground and surface water at 0.5 and 1.0 mg/L, respectively. General industrial wastewater discharge guidelines for manganese are no more than 1.0 mg/L [8]. Therefore, manganese as a pollution biomarker should be determined to assess environmental water quality to render managing water easier according to UN SDGs.

Flame atomic absorption spectroscopy (AAS) is used to analyze manganese. Inductively coupled plasma optical emission spectroscopy (ICP-OES) has received public favor because of its higher sensitivity than AAS [9]. Although high resolution ICP mass spectrometry (ICP-MS) has been introduced owing to a greater sensitivity than ICP-OES, it remains quite expensive [10]. Spectrometry is a conventional technique that is also used to determine manganese [11,12,13,14]. The techniques mentioned above may be associated with advanced technologies providing high accuracy, selectivity and sensitivity but require bulky instruments, specialized procedures and highly trained operators, time, cost and energy and are unsuitable for field surveys or onsite analysis. Alternative techniques to determine heavy metals have been developed to suit the onsite analysis applied to environmental monitoring [15,16,17]. Portable/deployable devices based on the electrochemical microfluidics technique proved to determine manganese concentration [18,19]. In addition, based on the absorbance-microfluidics, detection is also performed [20]. However, these alternative methods require specially designed equipment. Digital imaging, incorporating widely accessible smartphone cameras and image processing software, has recently become a choice of colorimetric analytical alternative sensing methods [21,22,23]. Using a digital color analyzer, “colors” are measured and converted into numerical values. Different color systems are utilized to construct a three-dimensional coordinate space, and the commonly used systems include Red Green Blue (RGB), Hue Saturation Value (HSV) and Gray models, where each color is represented by a single point. The color values of a single point are present as numerical values that can be used as analytical data [24]. The digital image colorimetric technique offers cost-effective sensing devices with the following characteristics: small, cheap, energy-saving, portable and independent of trained operators. By selecting a chemical reaction, a procedure can be developed for accuracy, precision, sensitivity, selectivity and sample throughput. The technique is useful to determine inorganic analytes such as calcium and magnesium in biodiesel [25] and organic species such as carbaryl residues in herbal medicines [21], formaldehyde in seafood [22] and antioxidant activity of Miang (fermented tea) [23]. The digital image colorimetric technique employing a scanner as a detector has been reported to determine manganese in water using mixed reagents 4-(2-pyridylazo)resorcinol (PAR) and poly(diallyldimethylammonium chloride) and a paper-based analytical platform [26].

Kinetic methods involving redox and catalytic reactions have been applied for chemical analysis [27,28]. Spectrophotometric determination of manganese using redox and catalytic reactions of 3,3′,5,5′-tetramethylbenzidine (TMB) has been reported [12,13,14]. However, in this work, sustainable downscaled catalytic colorimetric determination of manganese in freshwater using smartphone-based monitoring oxidation of TMB by periodate aims to provide a simple, smartphone-based colorimetric technique to determine manganese with great application potential for onsite environmental monitoring. The proposed procedure focuses on portable/deployable devices to achieve cost-effective and high throughput analysis concurrently with superior analytical characteristics, including sufficient effectiveness, sensitivity, selectivity, accuracy and precision. The proposed method was applied to determine manganese in canal and river water as an initial model for environmental samples.

## 2. Results

### 2.1. Reaction of Manganese (II)-Catalyzed Oxidation of TMB with Periodate

**Figure 1:**
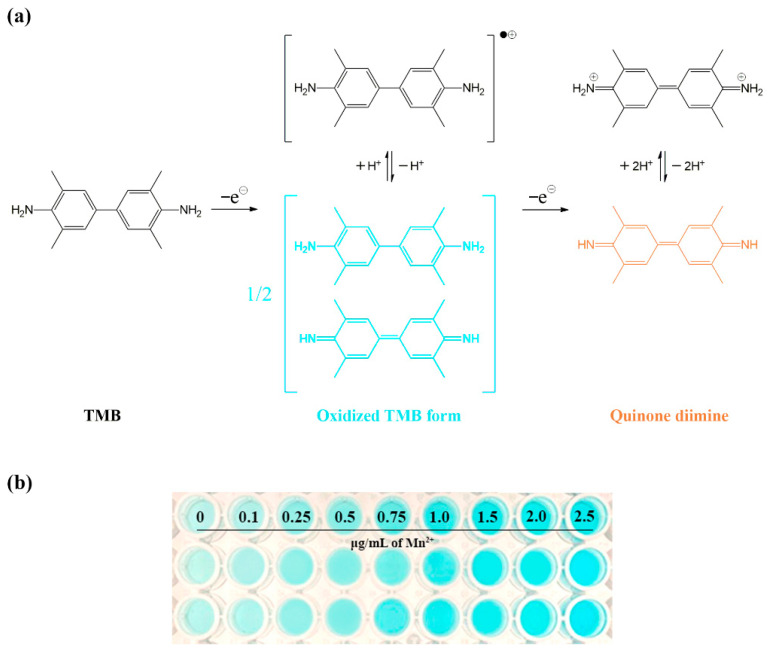
Oxidation of TMB by periodate using manganese (Mn(II)) as a catalyst: (**a**) the mechanism illustration and (**b**) the bluish-green products (oxidized TMB form) produced by being catalyzed with various Mn(II) concentrations (reaction condition: 2.08 mM TMB, 4.3 mM KIO_4_, phosphate buffer pH 6).

The oxidation of TMB using periodate with one-electron oxidation produces a bluish-green product, a meriquinoid complex comprising diamine (TMB) and diimine (quinone diimine), as illustrated in Figure 1a. An excessive concentration of periodate results in an orange product, most likely resulting from more extensive oxidation. One study, conducted on the effects of manganese concerning the rate of TMB oxidation by periodate, reported the catalytic effect of Mn(II). Although it remained unclear regarding insights into the catalytic reaction mechanisms, metal ions were likely to interact with the active center of the reaction rather than the initial species (TMB and periodate) [13]. This effect was employed to develop catalytic methods to determine manganese. The formation rate of the bluish-green product corresponds to the concentration of Mn(II)-catalyst [12]. The bluish-green product of oxidized TMB, formed by periodate with various manganese concentrations catalyzed in the developed procedure, is demonstrated in Figure 1b.

### 2.2. Optimum Buffer Condition

**Figure 2:**
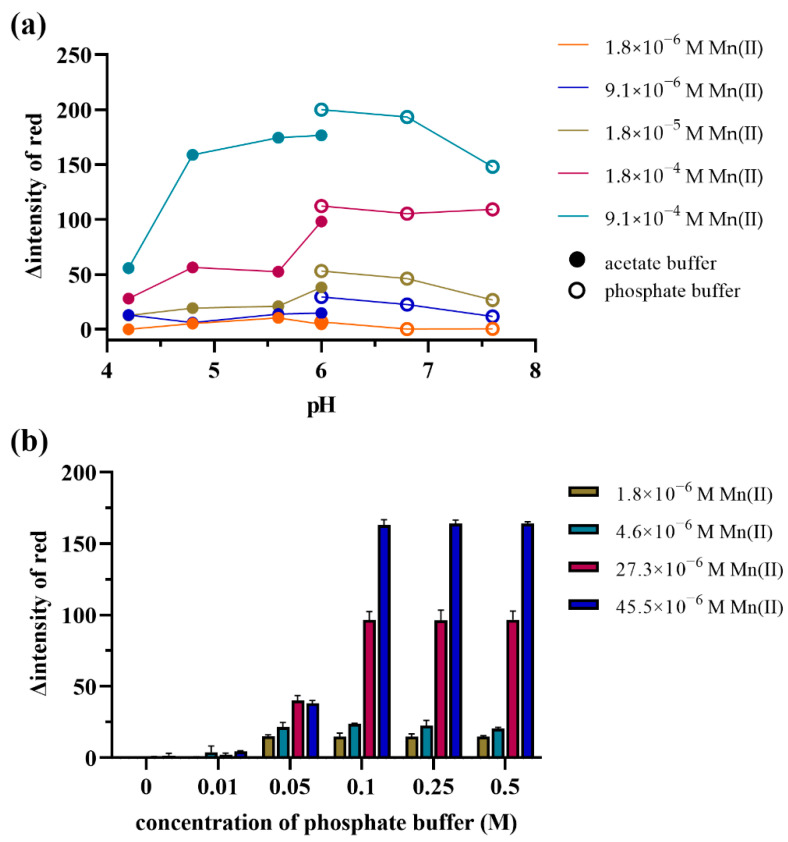
Oxidation of TMB by 4.3 mM KIO_4_ with various concentrations of Mn(II)-catalyzed (M) under: (**a**) different pH levels of 0.1 M acetate buffer and 0.1 M phosphate buffer conditions (**b**) different concentrations of phosphate buffer pH 6.

To observe optimum buffer condition for Mn(II)-catalyzed oxidation of TMB with periodate, the common acetate and phosphate buffers at different pH levels were investigated. A 0.1 M acetate buffer was varied at pH 4.2, 4.8, 5.6 and 6.0, and a 0.1 M phosphate buffer at pH 6.0, 6.8 and 7.6. The oxidation of TMB catalyzed by Mn(II) at five different Mn(II) concentrations (1.8 × 10^−6^, 9.1 × 10^−6^, 1.8 × 10^−5^, 1.8 × 10^−4^ and 9.1 × 10^−4^ M) was monitored at the various buffers stated above at 1 min, and the concentrations of TMB and periodate were fixed at 4.16 and 4.3 mM, respectively. The catalytic activity of the oxidation of TMB with periodate by Mn(II) was estimated compared with noncatalyzed by Mn(II). The catalytic activity was evaluated as a numerical figure derived from the color intensity of the bluish-green product of oxidized TMB (oxidized TMB form) and expressed in terms of the relative color intensity of red (∆intensity of red). The relative color intensity of red was the intensity of red when contrasted to a blank background, constituting the intensity of the product when noncatalyzed by Mn(II). The occurring bluish-green product differed when catalyzed using different concentrations of Mn(II). Figure 2a shows the catalytic activity of Mn(II) under the conditions of acetate buffer at pH 4.2, 4.8 and 5.6, rarely showing catalytic activity at low concentrations of Mn(II). Under conditions of phosphate buffer at pH 6.0, the lowest concentration (1.8 × 10^−6^, 9.1 × 10^−6^ and 1.8 × 10^−5^ M) of Mn(II) showed more catalytic activity than under others, meaning that it could provide an excellent limit of Mn(II) detection.

In order to investigate the impact of the concentration of phosphate buffer, the concentration of the phosphate buffer pH 6 was varied at 0.01, 0.05, 0.1, 0.25 and 0.5 M. The oxidation of TMB catalyzed by Mn(II) at four different Mn(II) concentrations (1.8, 4.6, 27.3 and 45.5 × 10^−6^ M) under 2.08 mM TMB, 4.3 mM KIO_4_ and different concentrations of phosphate pH 6 was monitored at 5 min. According to the results, the proposed mechanism cannot be produced under phosphate buffer conditions of 0 or 0.01 M. At 0.05 M of phosphate buffer conditions, the oxidation occurs at the same level with and without catalysis. This may involve complicated mechanisms. According to the proposed mechanism, the bluish green, oxidized TMB products were proportional to the concentration of Mn(II) under 0.1, 0.25 and 0.5 M phosphate buffers. No significant differences occurred among these buffer concentrations, as shown in Figure 2b.

### 2.3. Concentration of Potassium Periodate

**Figure 3:**
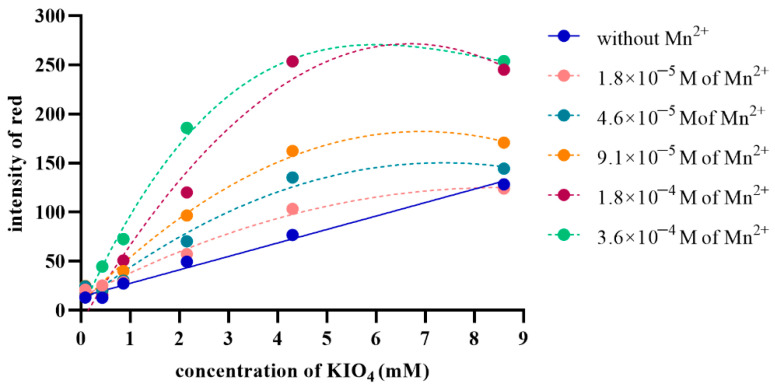
Intensity of the bluish-green, oxidized TMB products as a function of periodate concentration, compared across different Mn(II)-catalyst concentrations at 1 min (reaction condition: 4.16 mM TMB, 0.1 M phosphate buffer pH 6).

The influence of reagent concentration needs to be investigated because it affects the kinetics of oxidation. When the periodate concentration is appropriately fixed, the kinetics of TMB oxidation are noticeably determined by Mn(II) concentration. The concentrations of periodate were varied at 0.043, 0.086, 0.43, 0.86, 2.15, 4.3 and 8.6 mM to investigate the optimum concentration. The concentration of TMB was fixed at 4.16 mM and was oxidized under 0.1 M phosphate (pH 6) at 1 min. The bluish-green, oxidized TMB product was evaluated as a numerical number derived from the color intensity of red. The oxidation of TMB by periodate without Mn(II) catalysis was directly proportional to the concentration of periodate, as shown in the dense blue line in Figure 3. The oxidation was catalyzed by Mn(II), resulting in increased bluish-green, oxidized TMB products. The dotted lines in Figure 3 show increasing oxidized TMB products by increasing concentrations of Mn(II)-catalyzed, according to the related work by Beklemishev et al. [13]. Considering the oxidation at any concentrations of periodate, the bluish-green, oxidized TMB products were proportional to the concentration of Mn(II) at the concentrations of periodate of 0.86 to 8.6 mM. The linear relationship between the bluish-green, oxidized TMB product and the concentration of Mn(II) was taken ranging from 9.1 × 10^−6^ to 1.8 × 10^−4^ M at 0.86 and 2.15 mM of periodate, Mn(II) ranging from 0.1 to 10 μg/mL at 4.3 mM of periodate and Mn(II) ranging from 4.6 × 10^−6^ to 1.8 × 10^−4^ M at 8.6 mM of periodate as shown in Supplementary Materials Figure S1. Likewise, the greatest sensitivity was also found at 4.3 mM of periodate when considered from the slope of the calibration curve plotted between the ∆intensity of red and the concentration of Mn(II)) as shown in Figure S2, indicating that the optimum periodate concentration was 4.3 mM.

### 2.4. Concentration of 3,3′,5,5′-TMB

**Figure 4:**
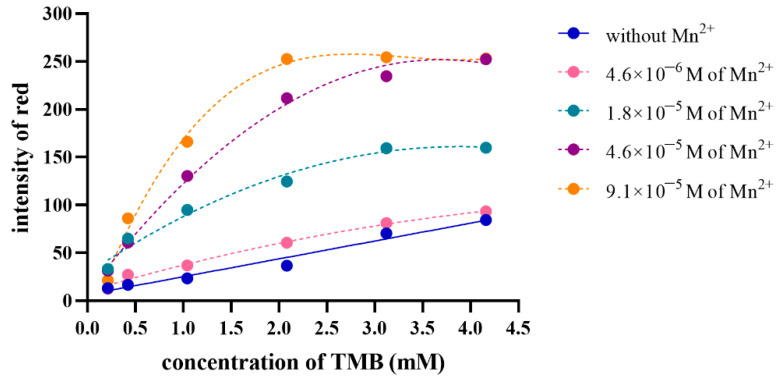
Intensity of the bluish-green, oxidized TMB products as a function of TMB concentration, compared across different Mn(II)-catalyst concentrations (reaction condition: 4.3 mM KIO_4_, 0.1 M phosphate buffer pH 6).

Another influence on the kinetics of oxidation is substrate concentration; thus, the concentration of TMB is optimized. TMB concentrations were investigated from 0.21 to 4.16 mM, while periodate concentration was kept constant at 4.3 mM. The oxidation of any concentrations of TMB was compared with and without various concentrations of Mn(II)-catalyst under 0.1 M phosphate pH 6 at 5 min. The color intensity of red was used to assess the value of the bluish-green, oxidized TMB product. As illustrated by the dense blue line in Figure 4, the oxidation of TMB by fixed periodate and without Mn(II)-catalyst was linearly proportional to the concentration of TMB. The oxidation of TMB catalyzed by Mn(II) continued more rapidly than no catalysis, as shown using the dotted lines in Figure 4. Considering the oxidation at any concentration of TMB, the bluish green, oxidized TMB products were proportional to the concentration of Mn(II) at the concentrations of TMB ranging from 1.04 to 4.16 mM. The linear relationship between the bluish-green, oxidized TMB product and the concentration of Mn(II) was taken ranging from 4.6 × 10^−6^ to 1.8 × 10^−5^ M at 1.04 mM of TMB, Mn(II) ranging from 1.8 × 10^−6^ to 4.6 × 10^−5^ M µg/mL at 2.08 and 3.12 mM of TMB, and Mn(II) ranging from 4.6 × 10^−6^ to 4.6 × 10^−5^ at 4.16 mM of TMB, as shown in Figure S3 indicating that a low detection limit can be provided at 2.08 and 3.12 mM of TMB. However, the catalytic activity of 1.8 × 10^−6^ M of Mn(II) at 2.08 mM of TMB was more visible than at 3.12 mM, as indicated by a higher intensity of red in the bluish-green, oxidized TMB product.

### 2.5. Incubation Time

The kinetic study was conducted in a well plate using the oxidation condition under 2.08 mM TMB, 4.3 mM KIO_4_ under 0.1 M phosphate buffer pH 6. The different concentrations of Mn(II) comprised 1.8 × 10^−6^, 9.1 × 10^−6^, 1.4 × 10^−5^, 2.7 × 10^−5^, 4.6 × 10^−5^, 9.1 × 10^−5^ M and 0 M (without Mn(II)). A multi-head (8) autopipette was used to handle solutions. After final delivery of the solution (see 3.2), photographs were taken every minute for the first five minutes and at 7, 10, 15, 20, 25 and 30 min. The photographs were evaluated for red intensity (due to the bluish-green, oxidized TMB product). A plot of the red intensity against time exhibits as a kinetic plot, as it shows the color changes per unit of time. From Figure S4, it can be seen that the greater the concentration of Mn(II), the greater the change in color intensity, i.e., the greater the change in concentration of bluish-green, oxidized TMB product, per unit time, due to catalytic effect. Regarding a particular time, the difference of the red intensity of a particular Mn(II) concentration and the red intensity due to that without Mn(II) (0 M Mn(II)), the delta red intensity value was plotted against Mn(II) concentration, leading to a calibration plot. A sample solution was treated in the same way. The Mn(II) concentration in the sample can be evaluated from the calibration plot. Figure S5 shows different calibration plots obtained from different reaction times. The sensitivity observed from the slope of the calibration curve ranging from the Mn(II) concentrations from 1.8 × 10^−6^ to 4.6 × 10^−5^ M indicated the best sensitivity at 5 min (as shown in Figure S6). This condition would allow a sample throughput of 324 samples/h/in a 96-microwell plate by having a series of 6 standards with 324 samples for duplication.

### 2.6. Analytical Characteristics

#### 2.6.1. Linear Range, Linear Regression Analysis, Detection Limit (LOD) and Quantitation Limit (LOQ)

The calibration curve was created using the standard manganese solution and the following reagents: (1) phosphate buffer pH 6.0, (2) 4.3 mM of potassium periodate and (3) 2.08 mM of TMB under optimal conditions. The linear calibration graph over the range of 1.8 × 10^−6^ to 4.6 × 10^−5^ M (0.1 to 2.5 μg/mL) was analyzed using a *p*-value < 0.05 showing the regression equation: y = 2.98x + 12.468 (where y is the ∆intensity of red, x is the concentration of Mn(II) (µM)) and R-square was 0.9953. The LOD and LOQ were estimated using 3.3σ and 10σ as the standard deviation of the intercept divided by the slope of the linear calibration graph, respectively. LOD and LOQ were 3.6 × 10^−6^ and 1.1 × 10^−5^ M (0.2 and 0.6 μg/mL), respectively. These characteristics demonstrated that the proposed method could be applied to real environmental samples containing low concentrations of Mn(II). Compared with related literature, based on image processing, the LOD and selectivity of the proposed method were excellent [29]. Although the sensitivity of the proposed method was slightly lower than that in the study reported by Muhammad-aree et al. (the LOD of 0.11 μg/mL) [30], the selectivity to Mn(II) of the proposed method was better in the case of interference from copper and zinc that commonly involve interfering ions. Compared with the high selectivity reported by Kamnoet [26], the high selectivity of the proposed method was likewise found, but the proposed method led to a greater recovery.

#### 2.6.2. Accuracy and Precision

To inspect accuracy and precision, percent recovery, repeatability and intermediate precision were investigated. Five concentrations of Mn(II), ranging from 0.8 to 2.5 μg/mL, were determined in triplicate to quantify percent recovery. The recovery rates ranged from 98% to 109%, with relative standard deviations (RSDs) less than 10%. Eight concentrations of Mn(II) were used to create a calibration curve in triplicate to evaluate repeatability and intermediate accuracy defined in terms of sensitivity. Triplicate measurements of three operations investigated the repeatability, one in the morning and the others in the afternoon, and 7% RSD was found. Triplicate measurements of three operations were investigated over three days for intermediate accuracy, and 9% RSD was found.

### 2.7. Selectivity

The selectivity of the proposed method for manganese was requisite, meaning other ions in natural water samples must not have affected the occurring signal, whether negative or positive errors. Cobalt, copper, ferrous, ferric, lead and zinc ions, which were reported to be found in contaminated water [31,32], were investigated for their effects. Cobalt is rarely found in contaminated water, and a concentration range of 0.05 to 0.1 μg/mL was reported. The concentration of copper reported was approximately 0.1 to 2 μg/mL and 0.5 to 35 μg/mL found in natural rivers and water samples surrounding industrially polluted areas. The highest iron level was discovered to be around 10 μg/mL. On average, lead concentration of 0.6 μg/mL was found in natural rivers, and 1.5 μg/mL at the maximum was found in water samples surrounding fertilizer factory areas. Natural rivers found lead levels of 0.005 to 0.2 μg/mL, whereas water samples near fertilizer factories contained average zinc concentrations of 1.5 μg/mL. From the information in this work, cobalt, copper, ferrous, ferric, lead and zinc ions in the concentrations of 0.001 to 0.1, 0.1 to 35, 0.1 to 10, 0.1 to 10, 0.1 to 2 and 0.001 to 2 μg/mL were investigated for their effects, respectively. The condition (2.08 mM TMB, 4.3 mM KIO_4_, 0.1 M phosphate buffer pH 6) was used to determine Mn(II), but instead of Mn(II), each of the metal ions of the concentration range was studied. At 5 min reaction time, the red intensity was observed to be the same value of the reaction product without having the metal ion, i.e., no statistically significant differences with a *p*-value of <0.05 using Dunnett’s multiple comparisons test (ANOVA) in triplicate runs.

### 2.8. Application

The developed procedure was applied to determine manganese concentration in eight water samples collected from three sources: the canals passing through a community area, farmland and natural water (Mae Klang River) in Subdistrict Municipality Chomthong, Chiang Mai, Thailand. Canal Nos. 1 to 4 passed through a community area with washing activities including gray and murky water. Canal Nos. 5 to 7 run through farmland and river No. 1 comprises freshwater and is not involved in any activity. The water samples were filtered to remove contaminants composed of muck, weeds and other materials using Whatman filter paper No. 1. Then they were filtered through a nylon syringe filter (0.45 μm, ID 25 mm, MACHEREY-NAGEL, Düren, Germany) before determination. The manganese contents in five water samples were compared with ICP-OES as a reference method [33], as summarized in Table 1. The detected manganese concentration showed no statistically significant differences at a confidence level of 95% (*t*-test).

## 3. Materials and Methods

### 3.1. Chemicals

All analytical-grade chemicals were used: acetic acid (glacial), cobalt chloride, copper sulfate pentahydrate, iron standard solution, lead standard solution, manganese standard solution, zinc standard solution and 3,3′,5,5′-TMB from Merck, Darmstadt, Germany. Potassium dihydrogen orthophosphate and di-potassium hydrogen orthophosphate were obtained from Ajax Finechem, Hindmarsh, Australia. Potassium periodate was obtained from Carlo Erba, Val de Reuil, France, and sodium acetate anhydrous was obtained from QRëC, Auckland, New Zealand.

### 3.2. Proposed Analytical Device Set-Up and Procedure for Manganese Ion Determination

The proposed downscaling in a micrometer-scale procedure was operated in a microplate with 96 wells (Corning, AZ, USA) using a multichannel pipette for reagent solution handling and a smartphone as a detector. The chemical admixture procedure to determine manganese consisted of the following four steps: (1) 230 µL of 0.1 M buffer, (2) 10 µL of the manganese standard solution or sample, (3) 10 µL of potassium periodate and (4) 50 µL of TMB in 0.5 M hydrochloric acid. The control comprised deionized water instead of the manganese standard solution or sample. The mixture solutions appeared as a bluish-green color of the oxidized TMB (see Figure 1b). The kinetics of oxidation were limited by the concentration of manganese as the catalyst. The produced bluish-green products were dependent on manganese concentration at the initial rate. The intensity of the bluish-green product was monitored using a smart device camera (Apple iPhone8, Zhengzhou, China) as a detector. The microplate was placed in a light-controlled housing made by a 3D printer (Flashforge 3D printer, Zhejiang, China) in our laboratory to reduce the interference of noisy light while taking a picture by smartphone. The intensity values of bluish-green products were transformed from a photograph via image processing using any software such as ImageJ in this study, which could be enabled on any computer.

### 3.3. Inductively Coupled Plasma Optical Emission Spectrometry (ICP-OES) as Reference Method

The trace manganese (Mn(II)) was determined based on standard methods for examining water and wastewater (method 3113B) using ICP-OES on the Agilent Technologies 5900 ICP-OES (Santa Clara, CA, USA) [33]. The sample was introduced to the ICP-OES system through Tygon-type PVC peristaltic pump tubes using a peristaltic pump at a flow rate of 2.5 mL/min. The power output of the ICP-OES system and the radio frequency generators were 1200 W and 27 MHz, respectively. The flow rates of nebulizer argon gas, the auxiliary argon gas and plasma argon gas were 0.7, 1 and 12 L/min, respectively. The emission line was 257.610 nm.

## 4. Conclusions

The sustainable downscaled colorimetric determination of manganese (Mn(II)) to a sustainable and green procedure combining a microwell plate platform with smartphone detection and image processing was successfully achieved. Under the discovered optimum conditions, the oxidation of TMB by periodate using manganese as a catalyst resulted in a bluish-green, oxidized TMB product (oxidized TMB form) proportional to the concentration of Mn(II). The proposed method provides results with excellent accuracy and precision. Furthermore, efficient sensitivity and selectivity showed the potential for water sample applications that could identify the toxicity of environmental water according to the requirements of Australian and New Zealand guidelines, Environment Agency (UK) and Water Environment Partnership in Asia. We succeeded in measuring Mn(II) in canals and river water. It proved less expensive, easy to use, portable and exhibited high sample throughput, thereby representing a cost-effective green chemical analysis that could be deployed in any environment or situation.

## Figures and Tables

**Table 1:** Obtained concentrations of manganese in water samples (*n* = 3) using the developed and reference methods.

Title 1	Concentration of Manganese		
	Developed Method		ICP-OES ^1^ (μg/mL)
	(µM)	(μg/mL)	
Canal No. 1	<LOQ (6.01 ± 0.55)	<LOQ (0.33 ± 0.03)	0.322
Canal No. 2	<LOD (2.91 ± 2.00)	<LOD (0.16 ± 0.11)	0.170
Canal No. 3	21.68 ± 1.64	1.19 ± 0.09	1.210
Canal No. 4	25.50 ± 0.73	1.40 ± 0.04	1.370
Canal No. 5	<LOD (2.73 ± 0.91)	<LOD (0.15 ± 0.05)	0.120
Canal No. 6	ND	ND	0.018
Canal No. 7	ND	ND	0.001
River No. 1	ND	ND	0.001

^1^ the reference method. ND: not detected.

## Data Availability

The data presented in this study are available on reasonable request from the corresponding author.

## Supplementary Materials

The following supporting information can be downloaded at: https://www.mdpi.com/article/10.3390/molecules27154841/s1, Figure S1. Calibration plots: the delta red intensity vs. concentration of Mn(II); the red intensity due to bluish-green, oxidized TMB product occurred from the oxidation of TMB using different concentrations of periodate: 0.86, 2.15, 4.3 and 8.6 mM.; Figure S2. The sensitivity (the slopes of the calibration plots) of Mn(II)-catalyst detection when using different concentrations of periodate.; Figure S3. Calibration plots (delta red intensity vs. concentration of Mn(II); the delta red intensity due to the bluish-green, oxidized TMB product from the oxidation using 4.3 mM periodate and with different TMB concentrations: 1.04, 2.08, 3.12 and 4.16 mM; Figure S4. Kinetic plots due to the oxidation of 2.08 mM TMB by 4.3 mM periodate with various Mn(II) concentrations under phosphate buffer at pH 6.; Figure S5. Calibration plots: the delta intensity of red (the bluish-green, oxidized TMB product) vs. Mn(II) concentration with different incubation periods.; Figure S6. The sensitivity (the slopes of the calibration plots in Figure S5) at different incubation time durations.

## Sample Availability

Samples of the compounds are not available from the authors.
