# Supplementary material for: Sustainable Downscaled Catalytic Colorimetric Determination of Manganese in Freshwater Using Smartphone-Based Monitoring Oxidation of 3,3′,5,5′-Tetramethylbenzidine by Periodate
Source: Molecules. 2022 Jul 28;27(15):4841. doi: 10.3390/molecules27154841 (PMC9369721; doi:10.3390/molecules27154841)
Supplement: Supplementary file 1 [file molecules-27-04841-s001.zip › molecules-1786490-supplementary.pdf]

molecules

MDPI

Supplementary Materials

Article

Sustainable Downscaled Catalytic Colorimetric Determination of Manganese in Freshwater Using Smartphone-Based Monitoring Oxidation of 3,3′,5,5′-Tetramethylbenzidine by Periodate

Sutasinee Apichai [1,2], Parichart Kummuntakoon [3], Thanawat Pattananandecha [1,2], Jakaphun Julsrigival [1,2], Kasirawat Sawangrat [1,2], Fumihiko Ogata [4], Naohito Kawasaki [4,5], Kate Grudpan [2,6] and Chalermpong Saenjum [1,2,*]

1 Department of Pharmaceutical Sciences, Faculty of Pharmacy, Chiang Mai University, Chiang Mai 50200, Thailand; sutasinee.apichai@gmail.com (S.A.); thanawat.pdecha@gmail.com (T.P.); jakkaphun@gmail.com (J.J.); kasirawat.s@cmu.ac.th (K.S.)

2 Center of Excellence for Innovation in Analytical Science and Technology for Biodiversity-Based Economic and Society (I-ANALY-S-T_B.BES-CMU), Chiang Mai University, Chiang Mai 50200, Thailand; kgrudpan@gmail.com

3 Department of Chemistry, Faculty of Science and Technology, Chiang Mai Rajabhat University, Chiang Mai 50300, Thailand; parichart10983@gmail.com

4 Faculty of Pharmacy, Kindai University, 3-4-1 Kowakae, Higashi-Osaka 577-8502, Japan; ogata@phar.kindai.ac.jp (F.O.); kawasaki@phar.kindai.ac.jp (N.K.)

5 Antiaging Center, Kindai University, 3-4-1 Kowakae, Higashi-Osaka, Osaka 577-8502, Japan

6 Department of Chemistry, Faculty of Science, Chiang Mai University, Chiang Mai 50200, Thailand

* Correspondence: chalermpong.s@cmu.ac.th; Tel +66-89-950-4227

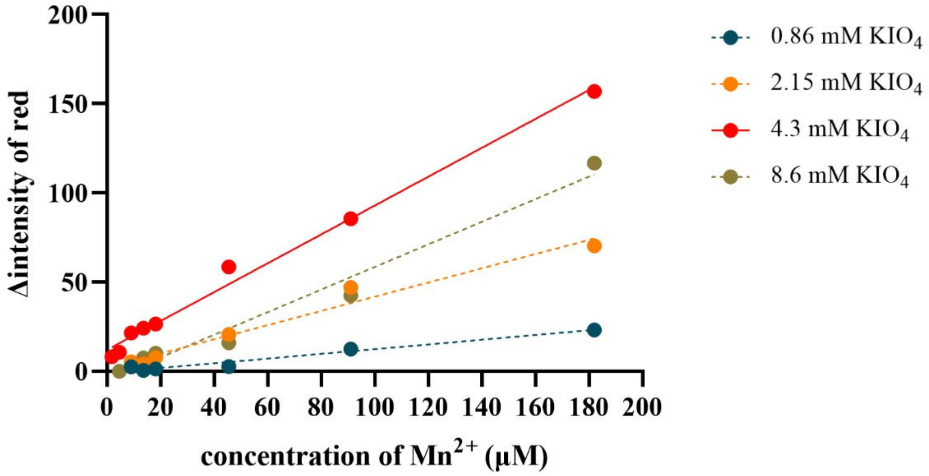

Figure S1. Calibration plots: the delta red intensity vs. concentration of Mn(II); the red intensity due to blu-ish-green, oxidized TMB product occurred from the oxidation of TMB using different concentrations of periodate: 0.86, 2.15, 4.3, and 8.6 mM.

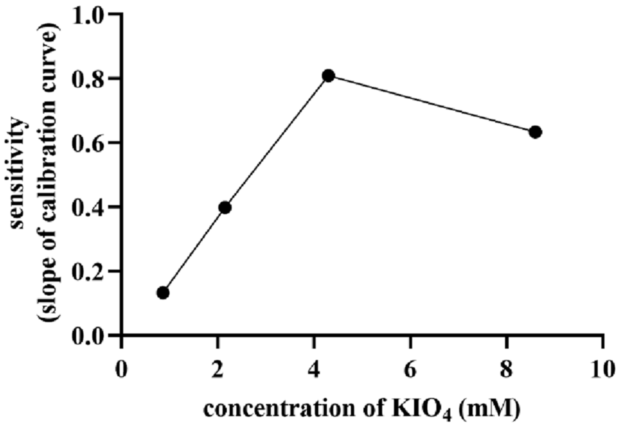

Figure S2. The sensitivity (the slopes of the calibration plots) of Mn(II)-catalyst detection when using different concentrations of periodate.

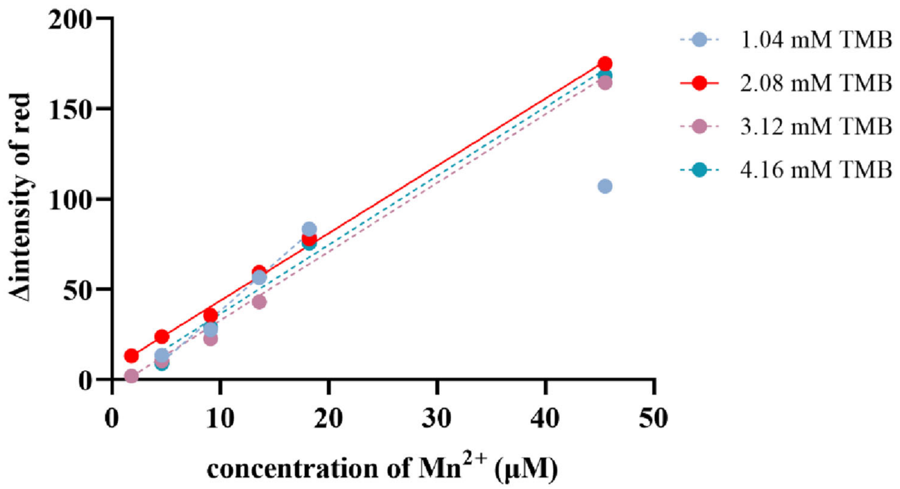

Figure S3. Calibration plots (delta red intensity vs. concentration of Mn(II); the delta red intensity due to the bluish-green, oxidized TMB product from the oxidation using 4.3 mM periodate and with different TMB concentrations: 1.04, 2.08, 3.12 and 4.16 mM.

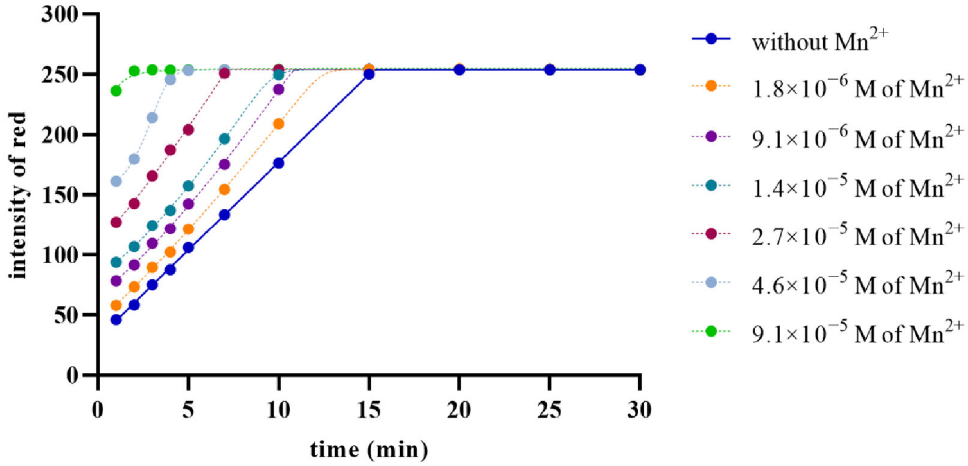

Figure S4. Kinetic plots due to the oxidation of 2.08 mM TMB by 4.3 mM periodate with various Mn(II) concentrations under phosphate buffer at pH 6.

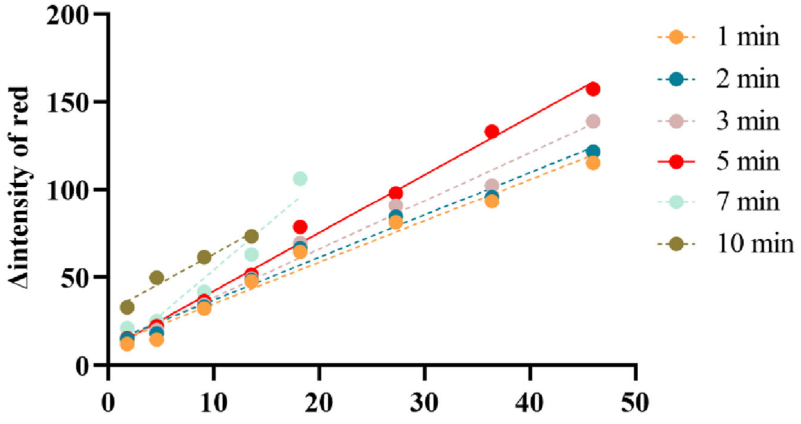

Figure S5. Calibration plots: the delta intensity of red (the bluish-green, oxidized TMB product) vs. Mn(II) concentration with different incubation periods.

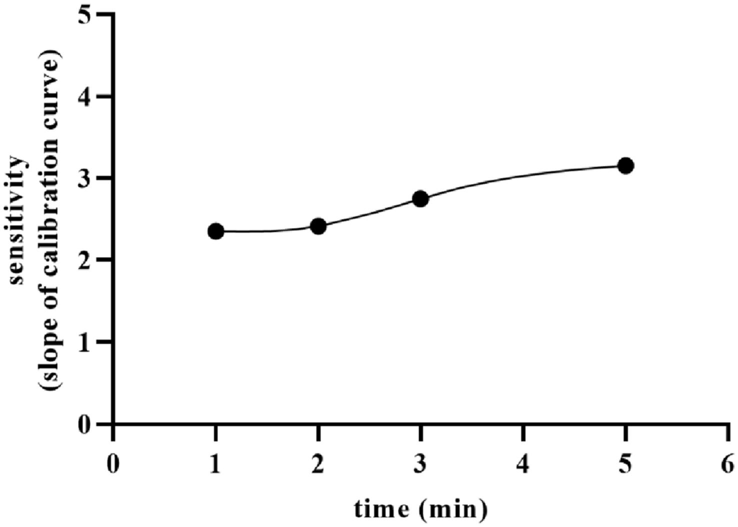

Figure S6. The sensitivity (the slopes of the calibration plots in Figure S5) at different incubation time durations.
